# Extended pancreas donor program – the EXPAND study rationale and study protocol

**DOI:** 10.1186/2047-1440-2-12

**Published:** 2013-07-01

**Authors:** Andrea Proneth, Andreas A Schnitzbauer, Florian Zeman, Johanna R Foerster, Ines Holub, Helmut Arbogast, Wolf O Bechstein, Thomas Becker, Carsten Dietz, Markus Guba, Michael Heise, Sven Jonas, Stephan Kersting, Jürgen Klempnauer, Steffen Manekeller, Volker Müller, Silvio Nadalin, Björn Nashan, Andreas Pascher, Falk Rauchfuss, Michael A Ströhlein, Peter Schemmer, Peter Schenker, Stefan Thorban, Thomas Vogel, Axel O Rahmel, Richard Viebahn, Bernhard Banas, Edward K Geissler, Hans J Schlitt, Stefan A Farkas

**Affiliations:** 1 Department of Surgery, University Hospital Regensburg, Franz-Josef-Strauss-Allee 11, 93053 Regensburg, Germany; 2 Department of General and Visceral Surgery, Goethe-University Hospital and Clinics, Theodor-Stern-Kai 7, 60590 Frankfurt am Main, Germany; 3 Centre for Clinical Studies, University Hospital Regensburg, Franz-Josef-Strauss-Allee 11, 93053 Regensburg, Germany; 4 Ludwig Maximilian's University, Department of Surgery, University Hospital Grosshadern, Marchioninistrasse 15, 81377 Munich, Germany; 5 Department of General and Thoracic Surgery, University Hospital Schleswig-Holstein, Arnold-Heller-Strasse 3, 24105 Kiel, Germany; 6 Department of Visceral, Thoracic and Vascular Surgery, University Hospital Marburg, Baldingerstrasse, 35043 Marburg, Germany; 7 Department of General, Visceral and Transplantation Surgery, Johannes Gutenberg University Mainz, Langenbeckstrasse 1, 55131 Mainz, Germany; 8 Department of Visceral, Transplantation, Vascular and Thoracic Surgery, University Hospital of Leipzig, Liebigstrasse 20, 04103 Leipzig, Germany; 9 Department of General, Thoracic and Vascular Surgery, University of Dresden, Fetscherstrasse 74, 01307 Dresden, Germany; 10 Visceral and Transplantation Surgery, Hannover Medical School, General, Carl-Neuberg-Strasse 1, 30625 Hannover, Germany; 11 Department of Surgery, University Hospital Bonn, Sigmund-Freud-Strasse 25, 53127 Bonn, Germany; 12 Department of Surgery, University Hospital Erlangen, Krankenhausstrasse 12, 91054 Erlangen, Germany; 13 Department of General, Visceral and Transplant Surgery, University Hospital Tübingen, Hoppe-Seyler-Str. 3, 72076 Tübingen, Germany; 14 Department of Hepatobiliary Surgery and Visceral Transplantation, University Medical Center Eppendorf, Martinistrasse 52, 20246 Hamburg, Germany; 15 Department of General, Visceral and Transplantation Surgery, Charité-University Medicine Berlin, Augustenburger Platz 1, 13353 Berlin, Germany; 16 Department of General, Visceral and Vascular Surgery, University Hospital Jena, Erlanger Allee 101, 07747 Jena, Germany; 17 Cologne-Merheim Medical Center, Department of Abdominal, Vascular, and Transplant Surgery, Witten/Herdecke University, Ostmerheimer Strasse 200, 51109 Cologne, Germany; 18 Clinic for General, Visceral and Transplantation Surgery, University Hospital of Heidelberg, Im Neuenheimer Feld 110, 69120 Heidelberg, Germany; 19 Department of Surgery, Knappschafts-Hospital, Ruhr-University Bochum, In der Schornau 23-25, 44892 Bochum, Germany; 20 Klinikum Rechts der Isar, Department of Surgery, Technical University of Munich, Ismaninger Strasse 22, 81675 Munich, Germany; 21 Department of General Surgery, University Hospital of Muenster, Albert-Schweitzer-Campus 1, 48149 Muenster, Germany; 22 Eurotransplant International Foundation, 2301CH, Leiden, The Netherlands; 23 Department of Internal Medicine II, University Hospital Regensburg, Franz-Josef-Strauss-Allee 11, 93053 Regensburg, Germany

**Keywords:** Pancreas transplantation, Organ allocation, Extended donor criteria, Rejection

## Abstract

**Background:**

Simultaneous pancreas kidney transplantation (SPK), pancreas transplantation alone (PTA) or pancreas transplantation after kidney (PAK) are the only curative treatment options for patients with type 1 (juvenile) diabetes mellitus with or without impaired renal function. Unfortunately, transplant waiting lists for this indication are increasing because the current organ acceptability criteria are restrictive; morbidity and mortality significantly increase with time on the waitlist. Currently, only pancreas organs from donors younger than 50 years of age and with a body mass index (BMI) less than 30 are allocated for transplantation in the Eurotransplant (ET) area. To address this issue we designed a study to increase the available donor pool for these patients.

**Methods/Design:**

This study is a prospective, multicenter (20 German centers), single blinded, non-randomized, two armed trial comparing outcome after SPK, PTA or PAK between organs with the currently allowed donor criteria versus selected organs from donors with extended criteria. Extended donor criteria are defined as organs procured from donors with a BMI of 30 to 34 or a donor age between 50 and 60 years. Immunosuppression is generally standardized using induction therapy with Myfortic, tacrolimus and low dose steroids. In principle, all patients on the waitlist for primary SPK, PTA or PAK are eligible for the clinical trial when they consent to possibly receiving an extended donor criteria organ. Patients receiving an organ meeting the current standard criteria for pancreas allocation (control arm) are compared to those receiving extended criteria organ (study arm); patients are blinded for a follow-up period of one year. The combined primary endpoint is survival of the pancreas allograft and pancreas allograft function after three months, as an early relevant outcome parameter for pancreas transplantation.

**Discussion:**

The EXPAND Study has been initiated to investigate the hypothesis that locally allocated extended criteria organs can be transplanted with similar results compared to the currently allowed standard ET organ allocation. If our study shows a favorable comparison to standard organ allocation criteria, the morbidity and mortality for patients waiting for transplantation could be reduced in the future.

**Trial registration:**

Trial registered at: NCT01384006

## Background

Patients with type 1 (juvenile) diabetes mellitus generally suffer from long-term complications, most of which are related to vascular disease. Associated nephropathy can lead to dialysis with its own major risk factors for cardiovascular disease and a low quality of life. Simultaneous pancreas kidney transplantation (SPK) or pancreas transplantation after kidney (PAK) are the only curative treatment options for type 1 diabetic patients with impaired kidney function [1,2]. The aim of SPK is to optimize blood sugar control, prevent retinopathy progression and reduce other cardiovascular diseases, thereby restoring life quality and length [3-5]. Indeed, if SPK can be done before the patient requires dialysis, the positive effects are even more dramatic [6,7]. For instance, progressive retinopathy is halted or even reversed after SPK [8] and PAK [9], and motor and autonomous nerve functions improve [10]. Pancreas transplantation alone (PTA) is restricted to patients with good renal function who suffer from brittle diabetes or frequent hypoglycemia; this is the only treatment option allowing non-uremic patients with brittle diabetes long-term insulin-independency.

Waitlists are constantly increasing in the Eurotransplant (ET) area. In 2000 there were 195 people on the waitlist for pancreas or islet cell transplantation, with a total of 331 transplantations performed in that year. In 2010 the waitlist increased to 337 patients, but only 249 transplantations were possible. Thus, time on the waitlist in Germany almost doubled within the last ten years and is now close to two years (data provided by ET), leading to a significantly worse outcome and higher morbidity for the patients. One major reason for this decline may be the fact that – with constantly increasing mean donor age - a large number of pancreas allografts potentially eligible for transplantation were excluded by the ET pancreas allocation system (EPAS) until December 2011. Those criteria excluded donors older than 50 years and those with a body mass index (BMI) ≥30 (ET Manual Version 26 May 2009). As the average age of a post-mortal organ donor in the ET area is now 58 years, donor selection by age has been a major factor contributing to the current donor pancreas shortage. In Germany (year 2009), the total number of deceased organ donors was 1,217. However, only 407 donors were at an acceptable age of < 50 years. Altogether, 594 patients were not even screened for pancreas donation due to age, regardless of all other medical conditions. In addition, approximately 13% of potential donors were not eligible for pancreas donation due to the BMI restriction (data provided by Deutsche Stiftung für Organtransplantation e.V. (DSO)). Moreover, in the ET-area pancreas grafts are allocated without local priority, frequently leading to rather long ischemic times due to transportation issues. In summary, because of the shortage of organ donors there is a critical need to use more of the available pancreas grafts for transplantation [11].

Currently, available retrospective data from centers outside of the ET area suggest a similar outcome after transplantation using extended criteria organs; however, there is no prospective controlled trial addressing this issue. Therefore, the aim of our multicenter trial is to investigate the hypothesis that organs from donors 50 to 60 years old or with a BMI higher than 30, using local allocation with shorter ischemic times, can be transplanted with similar results compared to the standard criteria organs. The potential benefit for the patient who agrees to be transplanted with an extended donor criteria (EDC) pancreas (simultaneously with a standard kidney from the same donor) is a reduced waiting time with increased survival and life quality. This could mean that patients will be free from dialysis and insulin therapy earlier than presently expected in this region. We suggest that all pancreas transplantation programs in Germany (later, in the ET area) will benefit from this allocation system, as these EDC allografts will be available in addition to the pancreas grafts allocated currently, thereby increasing the pool of organs for pancreas transplantation. If the EXPAND study shows that EDC pancreases can be used with a regional allocation system without additional risk, this will potentially lead to a readjustment of pancreas allocation towards a regional allocation and transplantation system, which might achieve better results for more pancreas transplant recipients.

## Methods/Design

### Objectives and study concept

**Figure 1:**
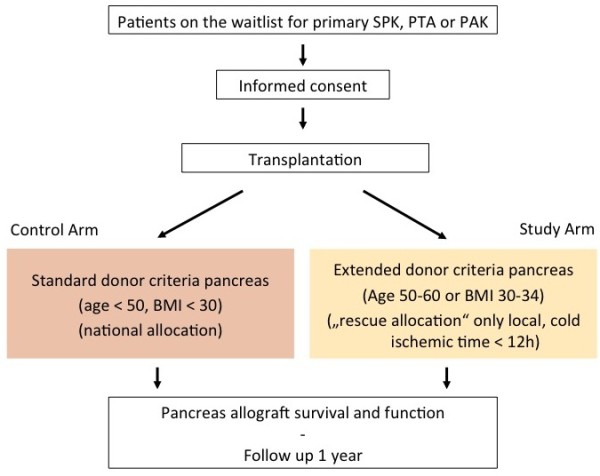
Inclusion scheme.

This study is a prospective, multicenter, single blinded, non-randomized, two-armed trial comparing outcome after SPK, PTA or PAK transplantation of organs with the currently allowed standard donor criteria to organs of donors with extended criteria. Extended criteria means a BMI 30 to 34 or donor age between 50 and 60 years. The enrollment phase will be three years with a follow-up period of one year. The primary endpoints are pancreas allograft survival and function at three months after transplantation. Our hypothesis is that pancreas organs of donors with extended criteria if transplanted regionally will have a similar function compared to organs with the currently allowed criteria. Overall survival, kidney and pancreas allograft function and survival, incidence of dialysis, post transplant insulin requirement, as well as infections and cardiovascular events will be assessed as secondary endpoints during the one-year follow-up. Secondary endpoints additionally include a quality of life assessment, biopsy proven acute rejections and time on the waitlist. In this study, patients who receive a standard criteria organ will be included in the control arm, and an equal number of patients who receive an EDC organ will be included in the study arm (Figure 1).

The EXPAND Study is an investigator-initiated trial for which the University Hospital Regensburg is the sponsor. Funding assistance is through grants provided by Astellas (Munich, Germany) and Novartis GmbH (Nürnberg, Germany).

### Allocation system

The allocation algorithm for potential deceased pancreas donors is regulated and defined in the ET handbook, section 7.1: Pancreas Allocation Algorithms. One reason for the decline in availability of pancreas allografts is the strict exclusion criteria set by the EPAS. As already mentioned, the main cut-off criteria for donors are age > 50 years or BMI ≥ 30 (current, standard criteria). For organs within the current criteria, allocation is performed according to HLA matching to the recipient, urgency status of recipient, waitlist time, and so on. Local distribution aspects are not part of the current allocation system, resulting in relatively long cold ischemic times in many cases.

For the EXPAND Study to allow allocation of EDC organs (age 50 to 60 or BMI 30 to 34) all responsible national and European authorities have been included in the development of an alternative rescue allocation system. ET and the German Medical Legislation Authorities, as well as the national Organ Procurement Organization (DSO) have been actively involved. In December 2010, ET established a rescue allocation system for extended criteria pancreas donors for the purposes of the EXPAND Study.

All deceased potential pancreas donors are screened for eligibility with regard to the current allocation al-gorithm. Organs meeting the standard criteria will be allocated to control arm patients. Other potential organs of donors with EDC who are between 50 and 60 years of age or have a BMI of 30 to 34 are now allocated according to the so-called ‘Beschleunigtes Vermittlungsverfahren’ (‘rescue allocation’), which has recently been implemented in the ET allocation system for extended pancreas use in Germany as part of this study. These organs are allocated only regionally to ensure a cold ischemic time of less than 12 hours. Other donor criteria are used to identify potential donors such as serum amylase or lipase, time on the ICU for the donor or use of vasoactive drugs; the risk/benefit assessment is made at the discretion of the transplant surgeons in both groups, with no distinct cut off line for those parameters.

Organ procurement and transplantation procedures are carried out according to internationally defined standards of organ procurement and pancreas transplantation. In Germany, arterial perfusion with histidine-tryptophan-ketoglutarate (HTK) solution is routinely performed and a backtable perfusion through the portal vein is recommended. In the case of an EDC organ, the regional allocation is performed with the following rules:

1. Regional centers receive organ offers in a rotation pattern. This means that every center receives (in a pre-defined fashion) sequential organ offers independent of the size of their waiting list or number of pancreas transplantations per year.

2. In case the first center rejects the offer, the organ is automatically offered to the procuring site.

The center receiving an offer can choose any (consented) recipient of the same ABO blood type, with a negative cross match, in their center for the transplantation. These rules aim for a fair local distribution, support small and very dedicated centers, and attempt to minimize cold-ischemic time.

### Study population

All patients on the waitlist for primary SPK, PTA or PAK meeting the inclusion criteria are eligible for the trial.

### Inclusion criteria

Adult patients >18 years of age eligible for primary SPK, PTA or PAK standard or extended criteria organ allocation are included in the study when informed consent is given prior to transplantation. In addition, the crossmatch testing must be negative.

### Exclusion criteria

Patients with malignant diseases within the past five years prior to transplantation (except for squamous cell carcinoma and basal cell carcinoma of the skin) are excluded. Patients who are listed for pancreas re-transplantation are excluded, as well as women of childbearing potential not willing to take contraception. Also, patients with a psychological, familial, sociological or geographic condition potentially hampering compliance with the study protocol and follow-up schedule, and patients under guardianship (for example, individuals who are not able to freely give their informed consent) are excluded.

### Consent

A study investigator will contact patients on the waitlist to introduce the study and associated procedures. An important part of the consent procedure is that the patient agrees to receive an extended criteria organ. When informed consent is given, patients are asked again for their consent at the time of transplantation. A patient is only eligible for an extended criteria organ when written and signed informed consent is given.

### Procedures to minimize bias

An important issue of this study was not only to confirm the safe use of EDC organs, but also to assess the quality of life experienced by the patient. To allow an unprejudiced evaluation, patients are blinded to their group inclusion. Because the transplant surgeon needs to consider all risk factors associated with the offered organ in relation to the patient, a double-blind set-up was not deemed appropriate. Additionally, randomization is not possible since inclusion into each study arm depends on the criteria (standard versus extended) of the organ, which is available for the patient at the time of transplantation.

### Interventions

#### Pre- and intraoperative data

Baseline data are documented, including demographics, medical history, current medication, liver and renal function, as well as history of renal replacement therapy and assessment of the quality of life. Intraoperative data (warm and cold ischemic times, blood-loss, requirement for blood products, incision to suture times) and donor data (age, serum-sodium, gamma- glutamyl transpeptidase, BMI, infectious status) are also documented.

#### Treatment regimen

Organ procurement and transplantation procedures are carried out according to internationally defined standards of organ procurement [12] and pancreas transplant procedures [1]. This trial is not aimed at establishing new immunosuppressive strategies, but rather to collect data prospectively in patients with EDC pancreas criteria, local allocation, and reduced cold-ischemic time in order to enlarge the donor pool for pancreas transplantation. To obtain comparable data, we strongly advocate the use of standardized immunosuppressive regimens in this study. We generally recommend the use of induction therapy with depleting or non-depleting antibodies [13]. Further maintenance therapy is normally performed with a combination therapy of tacrolimus (Prograf®, Astellas Pharma GmbH, Munich, Germany) (center specific trough levels), primarily MMF i.v. (CellCept®, Roche Pharma AG, Grenzach-Wyhlen, Germany) and later MPA oral (Myfortic®, Novartis Pharma GmbH, Nürnberg, Germany) (enteric-coated MPA). Steroids can be used according to center-specific protocols. To reduce pro-diabetic effects, early withdrawal of steroids (for example, two weeks after transplantation) is recommended [14]. Patients within the study will receive a combination immunosuppressive therapy of tacrolimus, MPA and steroids with early steroid withdrawal. Apart from this, all patients will be treated according to center specific standards. Transplant biopsies are not included within this protocol.

#### Follow-up

After transplantation, all patients will have follow-up study visits at days 7 and 14, and after 1, 3, 6, 9 and 12 months. Data collected in this study include only clinical and laboratory results that are routinely recorded anyway; investigations and examinations will not exceed the normal clinical standard at the participating sites. Documentation includes data on graft survival, graft function (laboratory values), incidence of clinically-diagnosed and biopsy-proven acute rejection (kidney and/or pancreas), severity of rejection (histological grade), changes in glucose metabolism, insulin requirement, need for dialysis, drug adverse events, infectious complications, cardiovascular incidents, general physical condition, and patient survival. At day 0 and at months 3, 6 and 12, a quality of life assessment will be performed (standardized questionnaire SF-12). During recruitment and follow-up of patients, regular monitoring of safety and endpoint data is performed according to good clinical practice (GCP) guidelines.

### Clinical sites

The study is planned as a multicenter (up to 20 sites) trial within Germany. As transplantation numbers in Germany are low for single centers, multiple sites are necessary to achieve the required patient number within a reasonable amount of time.

### Safety aspects

The Ethics Committee of the University Regensburg approved the study protocol on 17 December 2010 (number 10-101-0243), with approval following for all other participating sites. The study complies with the Declaration of Helsinki and the principles of GCP guidelines. Informed consent is obtained from each patient in written form while being on the waitlist and again prior to transplantation. A medical doctor familiar with the study informs the patient about the nature, duration and possible consequences of the trial. Patient safety and all potential threats for the patients are monitored once a year by an independent data monitoring committee. Qualified personnel at the sponsor site continuously review safety data, including adverse events and serious adverse events.

### Sample size calculation

The sample size was calculated by means of the primary endpoint, pancreas allograft survival rate after three months, on the assumption that a rate of 80% can be expected [15,16] whereas, the minimal accepted survival rate is 65%. The limit of 65% is based on the maximally acceptable lower organ survival rate, as agreed by the clinical trial centers. The significance level of alpha (one-sided) was set to 0.05 and beta was set to 0.20. This is in accordance with the estimation of a one-sided 95% confidence interval for the survival rate after three months. Assuming that a one-sided, binomial hypothesis test with a target significance level α = 0.05 and a target power 1−β = 0.80 will be used for analysis, 55 patients with an extended donor pancreas allograft should be enrolled in this study. A sample size of 55 patients with extended donor pancreas allografts achieves 80% power to distinguish between the two proportions 65% (p_0_) and 80% (p_1_) using a one-sided, binomial hypothesis test with a target significance level of 0.05. The control group should be of equal size for analyzing the secondary endpoints. Assuming a maximal dropout rate of 10%, a total of 120 patients will be required. Sample size was estimated based on exact binomial probabilities and calculation was performed using NCSS-PASS 2000. No power analysis for secondary endpoints was done.

### Statistical analysis

A one-sided, binomial hypothesis test with a target significance level α = 0.05 and a target power 1−β = 0.80 will be used for analysis of the primary endpoint. The primary analysis will be based on the intention-to-treat (ITT) analysis set. However, a sensitivity analysis will be done on a per-protocol (PP) analysis set. The latter serves to assess the robustness of the results. All safety data will be analyzed by means of the safety population. For the secondary endpoints the treatment group will be compared to the control group, and all statistical tests will be two sided and will be done at the 0.05 significance level. The exact analyzing strategies of all secondary endpoints will be further specified in a Statistical Analysis Plan, which will be finalized prior to database lock. The statistical analysis will be done by the Center for Clinical Studies at the University Hospital Regensburg.

### Current status

The first center was initiated in July 2011 and the first patient was enrolled on 26 July 2011. The last two centers have been initiated in January 2013. Of all 20 German centers, 12 centers are actively recruiting patients by now.

## Discussion

A recent single center study showed a 20-year survival rate of 58% after SPK; in comparison, the 20-year survival rate was 0% for patients who stayed on insulin therapy and dialysis [1]. An analysis of the Organ Procurement and Transplantation (OPTN) registries including 12,478 patients also reveals remarkable advantages of SPK transplantation. Data showed only a 58% four-year survival rate for patients on the waitlist, compared to a four-year survival rate of 90% after SPK [17]. To put this in perspective, the survival time on the waiting list is worse than for patients with stage 3 colon cancer [18]. Therefore, all patients with type 1 diabetes and impaired kidney function should be transplanted as soon as possible.

With this stark realization in times of organ shortage, new strategies to expand the donor pool are needed. Success in this regard has been realized with other organ transplants. For instance, the use of ‘marginal’ or ‘extended’ criteria donor organs is well established now, such as the so-called European Senior Donor Program for kidneys. Here, the potential higher risk associated with an older organ was compensated by the benefit of a short ischemic time through a change in the allocation system to only regional allocation [19,20]. Regarding pancreas transplantation, there are different factors that have to be considered when trying to expand the donor pool. There are data from other allocation areas throughout the world (United Network for Organ Sharing (UNOS) or UK) that a good overall outcome after pancreas transplantation can be achieved with donors exceeding the standard allocation criteria as currently defined by ET, if the cold ischemic time is kept short. In Great Britain, a local retrieval and allocation system was established in 2000 [21], resulting in a steep increase in pancreas transplantation [22,23] via extensive use of extended pancreas donors. These extended criteria avoid the donor BMI restriction and accept donors with an age between 8 and 68 years. Meanwhile 350 SPK and PTA were transplanted with an excellent one-year pancreas survival of 91%, kidney survival of 95% and a patient survival of 96% [24].

A retrospective analysis of OPTN registries in the US compared 8,850 SPK from young donors versus 776 SPK from donors > 45 years. The analysis showed that patients transplanted earlier using an organ from an older donor have similar survival rates when compared to those recipients waiting for a young donor for more than 1.5 years [25]. Good results were also achieved with extended and older pancreas donor organs in the US [16,26-30]. However, it is important to note that these data are only available from retrospective database analyses. Nonetheless, to keep ischemia times short, high volume centers outside of the ET zone use a regional allocation system [1-3,31-33].

For Germany, the existing allocation system has led to long ischemic times due to transportation issues; this fact is reflected in poorer outcomes in Germany [34,35] compared to high volume centers in the UK [24], USA [1,3,36] and Italy [15,32]. To address this problem, with the EXPAND study we are aiming to obtain prospective high-level evidence for changing our allocation system to regional-based allocation. It is expected that the potential higher risk of delayed graft function and pancreatitis of EDC organs [37] will be compensated by reduced ischemic times. The EXPAND study was developed with the aim of utilizing otherwise discarded EDC organs through this allocation strategy, leading to a significantly improved overall outcome in the ET zone. Moreover, this is the first study to evaluate the results of transplantation of EDC pancreas organs in a prospective clinical trial.

Interestingly, we can already report that after implementation of the rescue allocation system, transplant centers throughout Germany started to use EDC organs. This has led to an increase in transplant numbers - as had been anticipated when designing the study. Importantly, however, this does not mean that the quality of the transplanted organs and the success of this strategy are assured. This is what we expect to determine in our current trial and what the authorities want to see before extending the rescue allocation system to the whole ET area.

## Conclusions

The EXPAND study is the first prospective trial comparing and evaluating the outcome of standard criteria deceased donor pancreas organs to extended criteria organs. If our hypothesis, that EDC organs can be used with a similar outcome without additional risk for the patient is correct, the pancreas donor pool will successfully be expanded in the ET region. This will lead to more transplantations and shorter times on the waitlist with significantly reduced morbidity, increased life expectancy and better quality of life for many patients.

## Abbreviations

BMI: Body mass index; DSO: Deutsche Stiftung Organtransplantation; EDC: Extended donor criteria; EPAS: Eurotransplant pancreas allocation system; ET: Eurotransplant; MMF: Mycophenolate moffetil CellCept; MPA: Mycophenolic acid Myfortic; OPTN: Organ Procurement and Transplantation; PAK: Pancreas after kidney transplantation; PTA: Pancreas transplantation alone; SPK: Simultaneous pancreas and kidney transplantation.

## Competing interests

HJS has received lecture honoraria and reimbursement of travel expenses from Novartis, Astellas, Pfizer and Roche. He receives research support from Pfizer and Novartis. SF has received lecture honoraria, reimbursement of travel expenses and receives research support from Novartis, Astellas, BMS and Roche. All other authors declare that they have no competing interests.

## Authors’ contributions

AP^1^ contributed to launching and designing the study, coordination, writing the protocol and manuscript as well as data collection and inclusion of study patients and medical monitoring. AAS was involved in launching and designing the study, writing the protocol and manuscript and critical intellectual discussion of the manuscript as well as data collection and inclusion of study patients. FZ is the study statistician. JRF was involved in writing the protocol, local ethics approval and data monitoring. IH contributed to study coordination, local ethics approval and data monitoring. HA, PS^18^ and RV were involved in launching and designing the study and critical intellectual discussion of the manuscript. WOB, TB, CD, MG, MH, SJ, SK, JK, SM, VM, SN, BN, AP^15^, FR, MAS, PS^19^, ST, TV and BB were involved in data collection and inclusion of study patients, local ethics approval and critical intellectual discussion of the manuscript. AOR was involved in launching the study and in implementation of the rescue allocation system. EKG and HJS were involved in study design and coordination, ethics approval and critical intellectual discussion of the manuscript. SAF initiated the study concept and ethics approval, and was involved in writing the protocol and manuscript. All authors read and approved the final manuscript.
